# Seasonal Dynamics of Tick Species in the Ecotone of Parks and Recreational Areas in Middlesex County (New Jersey, USA)

**DOI:** 10.3390/insects14030258

**Published:** 2023-03-05

**Authors:** Julia González, Dina M. Fonseca, Alvaro Toledo

**Affiliations:** Center for Vector Biology, Department of Entomology, Rutgers University, 180 Jones Ave, New Brunswick, NJ 08901, USA

**Keywords:** ticks, ecotone, *Haemaphysalis*, *Ixodes*, *Amblyomma*, *Dermacentor*

## Abstract

**Simple Summary:**

People using forested parks for recreation often spend most time in grassy paths or meadows. These transitional zones are referred to as “ecotones”. In this study, we monitored the seasonal dynamics of questing ticks in forest/path and forest/meadow ecotones in five areas in Middlesex County, New Jersey. We found anthropophilic species such as *Ixodes scapularis*, *Amblyomma americanum*, and *Dermacentor variabilis* coexisting with *Haemaphysalis longicornis*, an invasive tick species first detected in NJ in 2017. Surveillance was conducted weekly from March to November 2020, and collected ticks were identified. The most abundant tick species was *H. longicornis*. The presence of anthropophilic ticks suggests the need for specific control approaches to target these habitats. In addition, the extraordinarily high numbers of *H. longicornis* collected in ecotones highlight the importance of monitoring its expansion due to its potential as a vector of animal and human diseases.

**Abstract:**

People often use parks and other forested areas for outdoor activities such as hiking and walking their dogs. Areas of primary use are paths or grassy meadows on the edges of the forests that constitute transitional areas between different plant communities (aka ecotones). In this study, we monitored the seasonal dynamics of questing ticks in forest/meadow and forest/path ecotones in five areas in Middlesex County, New Jersey (NJ). We found anthropophilic species such as *Ixodes scapularis*, *Amblyomma americanum*, and *Dermacentor variabilis* coexisting with *Haemaphysalis longicornis*, an invasive tick species first detected in NJ in 2017. Surveillance was conducted weekly from March to November 2020, and collected ticks were identified. The most abundant tick species was *H. longicornis* (83%), followed by *A. americanum* (9%), *I. scapularis* (7%), and *D. variabilis* (<1%). The seasonal dynamics of *A. americanum* and *I. scapularis* in the ecotone were similar to previous surveys in forest habitats. The presence of anthropophilic ticks, particularly *I. scapularis*, suggests the need for specific control approaches to target these habitats. In addition, the extraordinarily high numbers of *H. longicornis* collected in ecotones (1.70 ticks/m^2^) and frequent reports of this species on dogs highlight the importance of monitoring its expansion due to its potential as a vector of animal and human diseases.

## 1. Introduction

Tick-borne diseases continue to be a public health concern in the USA as the number of cases has increased in recent years [1,2]. The ongoing expansion of *Ixodes scapularis* Say and the increase in abundance of *Amblyomma americanum* (L.) throughout the Northeast and upper Midwest, as well as the recent detection of the invasive tick, *Haemaphysalis longicornis* Neumann, underscores the risk of tick-borne diseases in the northeastern USA [1,3,4]. Integrated tick management requires a good understanding of tick ecology [5], in particular, seasonal tick dynamics and habitat range, which can change due to multiple parameters, including the effects of climate, host communities’ movements, and human alteration of the landscape [6]. Tick surveillance estimates the density of host-seeking ticks [7] and is critical for monitoring temporal shifts and habitat ranges [5].

Different tick species can be found in different habitats (forests, grasslands, and meadows) depending on their host’s preference and ability to cope with temperature and humidity, which are impacted by canopy, shrub, and leaf litter composition [8,9,10,11]. For example, *Ixodes scapularis* is often most abundant in hardwood forests, while *A. americanum* is active in drier and warmer conditions than those tolerated by sympatric *I. scapularis* populations [9,10].

Ecotones are transitional areas between two environments that harbor specific arthropod communities [12,13]. While in New Jersey (NJ) *A. americanum* is also associated with forests and woodlands, this species as well as *D. variabilis* can thrive in open habitats, such as meadows and ecotones [10]. These areas have higher temperatures and lower relative humidity than forests due to the lack of trees and leaf litter that buffer extreme temperature and moisture conditions in forested areas [14].

Forest–grass ecotones, particularly those modified or originated by human activity, such as public parks and recreational areas, play an essential role in the enzootic cycle of infectious diseases, including zoonotic and vector-borne diseases, and have been associated with their re-emergence [15,16,17]. New Jersey has a high incidence of tick-borne diseases [18]; however, it lacks statewide surveillance to monitor tick species distribution and abundance, which are critical to implementing tick control strategies. This study aims to fill this gap by examining the abundance and seasonality of tick species in the ecotone of public parks and recreational areas to examine the likelihood of tick-risk encounters for humans and pets.

## 2. Materials and Methods

### 2.1. Study Area

**Figure 1:**
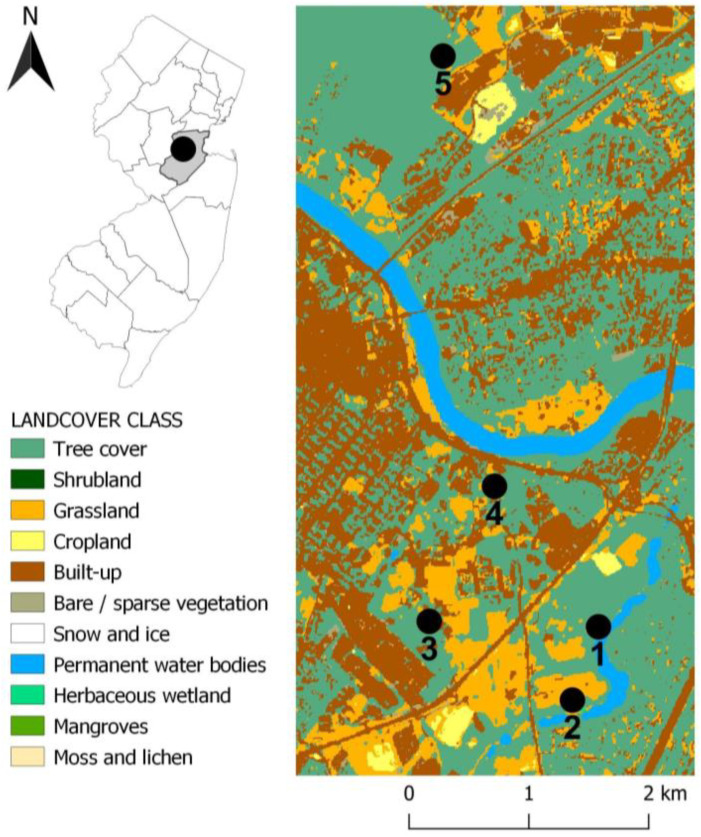
Map of sampling sites in the study area (Middlesex, NJ). QGIS map base ESA World Cover [19].

This study was conducted in five New Brunswick and Piscataway public parks and recreational areas within the Rutgers School of Biological and Environmental Sciences and the NJ Agricultural Experiment Station (Figure 1 [19]). The sites included deciduous forests dominated by oak and maple trees and huckleberry and blueberry shrubs [20]. Site 1 was within a botanical garden composed of small forest patches near the Raritan river; site 2 was located in one of Rutgers’ horticultural research farms; site 3 was composed of small forest fragments and grass meadows used for experimental goat and sheep rearing; site 4 was a meadow and wooded park associated with a conference center; and site 5 was an ecological forest preserve with a network of trails. At each site, we sampled three 20 m transects delimitated by permanent flags and arranged along the ecotone border. The vegetation between the mowed lawn or paths and the forest comprised low brushes and mixed grasses.

All sites were in Middlesex County (NJ), where the annual average temperature is 13 °C (minimum 8 °C and maximum 19 °C), and the annual total precipitation is 49 inches [21].

### 2.2. Tick Surveillance

Tick sampling was conducted weekly from mid-March until the end of November 2020. Although *Ixodes scapularis* adults were still active in December–February, based on preliminary surveys, the period chosen is when overall tick abundance was highest. Sampling was performed between 0900 h and 1600 h using a white crib flannel sweep measuring 50 × 100 cm with a PVC pipe handle [22]. Each transect was sampled in 2 m intervals because *H. longicornis* does not attach firmly to the flannel and often drops off over longer intervals [23]. Ticks were collected from both sides of the sweep and morphologically identified in the laboratory to species using a stereomicroscope (Leica S8 APO, Leica Microsystems) following appropriate taxonomical keys [24,25,26]. Tick numbers by life stage and species were recorded for each site. In addition, temperature and relative humidity data from a close-by weather station (Society Hill, NJ) were registered for each site right before sampling to determine the potential effects of environmental conditions on tick abundance. The observation of extreme shifts in local measures of temperature and relative humidity depending on where the measuring device was placed (for example, wind direction, recent rain event, patches of sun, etc.) led us to use data from the weather station instead of direct measurements using hand-held devices.

### 2.3. Data Analyses

We calculated relative abundance of ticks as the number of ticks collected per meter. We used non-parametric Kruskal–Wallis test and Dunn’s multiple comparisons tests to evaluate differences in abundance between sites. We correlated the periods of maximum tick abundance or peaks by species and life stage with temperature and relative humidity using non-parametric Spearman rank correlations. The correlation between tick abundance and saturation deficit (SD) was also examined. Saturation deficit is the atmosphere’s drying power and combines temperature and relative humidity data according to the formula [27]:

SD = (1 − RH/100) × 4.9463e^0.0621T

Finally, a linear regression was used to define the relationship between those correlated variables (maximums of relative tick abundance and temperature, relative humidity, or SD). For all tests, the level of statistical significance was *p* = 0.05. All statistical tests were performed using GraphPad Prism version 8 [28].

## 3. Results

We collected a total of 10,884 ticks of 4 different species, including *H. longicornis*, *A. americanum*, *I. scapularis*, and *D. variabilis*. The most abundant tick species was *H. longicornis* (83.1%), followed by *A. americanum* (9.5%), *I. scapularis* (7.3%), and *D. variabilis* (0.1%) (Table 1). All life stages were collected for all identified species except for *D. variabilis*, for which adults were the only stage found. Larvae were the most abundant stage for *H. longicornis*, *A. americanum*, and *I. scapularis*, representing 89%, 86%, and 81% of the total number of species, respectively. Nymphs and adults represented 9% and 2% of the total tick number for *H. longicornis*, 9% and 5% for *A. americanum*, and 5% and 13% for *I. scapularis* (Table 1).

Overall tick abundance and the abundance of *A. americanum* did not differ among sites; however, site 5 had a higher abundance of *H. longicornis* than site 1, and site 4 had a lower abundance of *I. scapularis* than site 3 (Table 2). The number of *D. variabilis* recorded was not sufficient to assess statistical differences between sites. To increase the statistical power when analyzing the effect of environmental variables on each tick species and life-stage abundance, we pooled data from the five sites.

**Figure 2:**
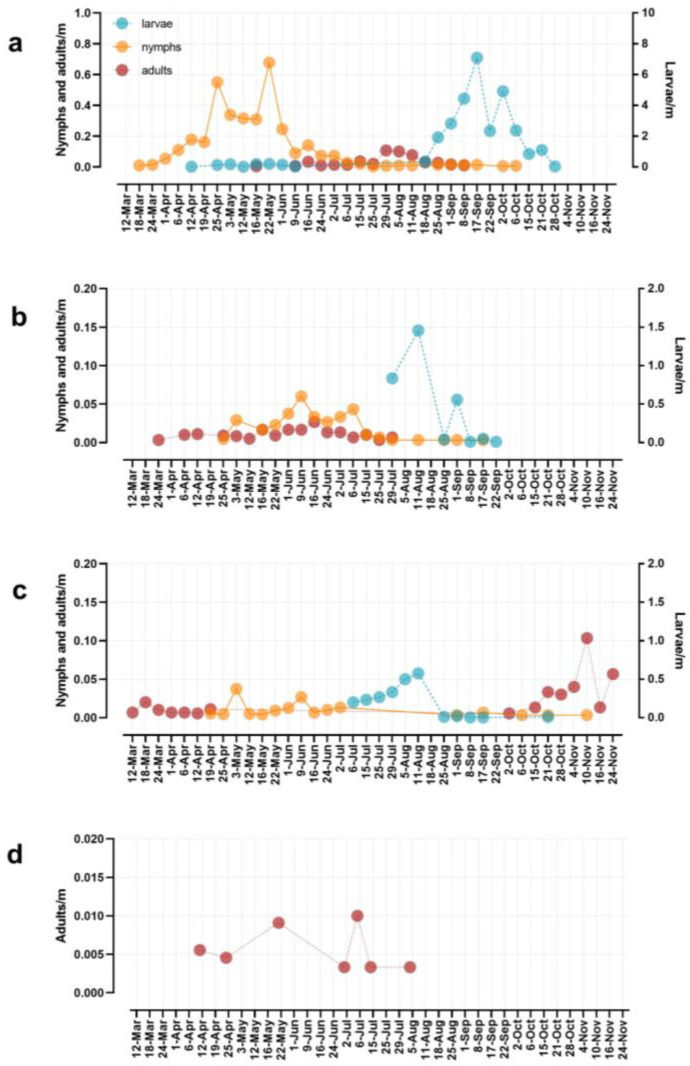
Seasonal dynamic of each life stage and tick species collected. Samplings with zero ticks collected are not represented. (**a**) *Haemaphysalis longicornis*; (**b**) *Amblyomma americanum*; (**c**) *Ixodes scapularis*; (**d**) *Dermacentor variabilis*. Notice the difference in scale (5× higher) for *H. longicornis*.

*Haemaphysalis longicornis* was the dominant species in the ecotone from late March to late October (Figure 2), except in July when coinciding with their respective peaks of larval activity, *I. scapularis* and *A. americanum* were the most abundant species. After the end of October, only *I. scapularis* adults were found (Figure 2).

**Figure 3:**
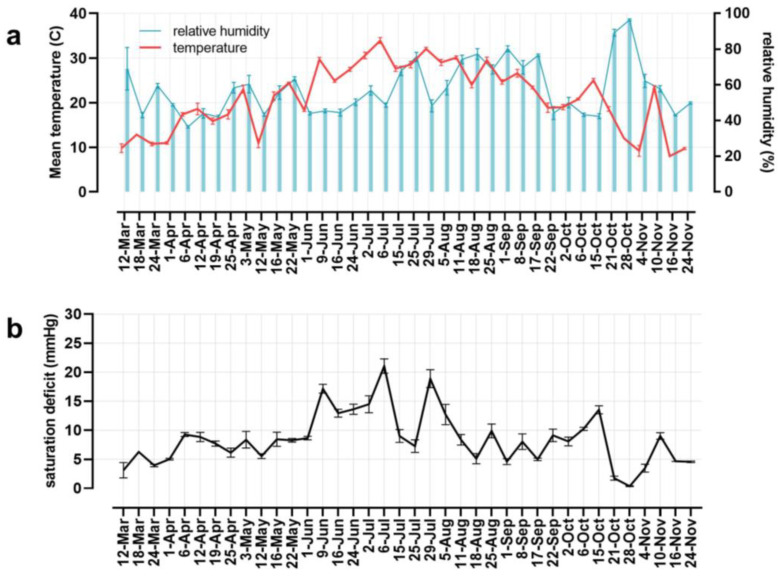
(**a**) Mean temperature and relative humidity calculated from the data registered in the weather station (Society Hill, NJ) at the start time of the tick sampling in each site (n = 5/day). (**b**) Saturation deficit calculated combining the data of temperature and relative humidity registered (more details in Materials and Methods).

**Figure 4:**
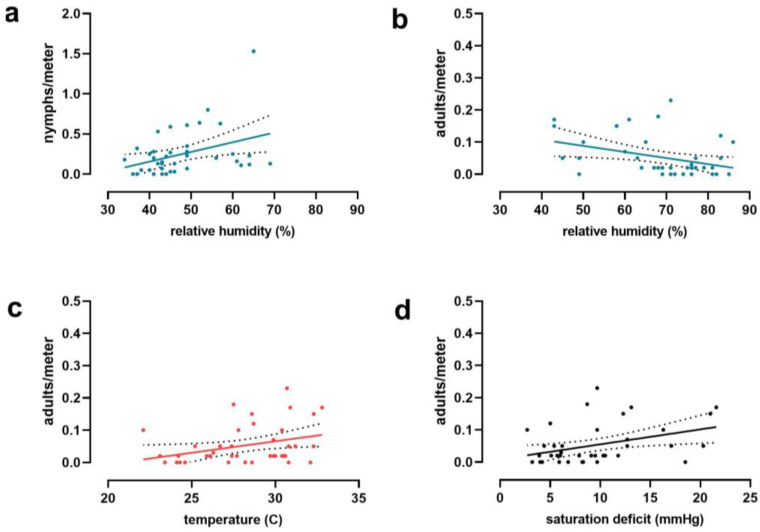
Representation of the linear regression equations between the environmental variables registered and the relative abundance of *Haemaphysalis longicornis* activity peaks of nymphs (**a**) and adults (**b**–**d**).

Nymphs of *H. longicornis* were the first stage detected in the ecotone of deciduous forests in Middlesex (NJ) and were present from 18 March to 6 October. Adults were present in the ecotone from mid-May to the beginning of September, overlapping with nymphs and larvae in summer (Figure 2a). Larvae were collected in small numbers between 12 April and 9 June, but their activity peaked from mid-August until the end of October. The peak of nymphal activity ranged from mid-April to the first week of June when the temperature increased (Figure 3a). Moreover, the highest SD values were registered between June and August (Figure 3b), when adult activity peaked, and the number of nymphs decreased (Figure 2a). Indeed, the abundance of adults was correlated with temperature, relative humidity, and saturation deficit (Table 3). Temperature and SD were linearly related to *H. longicornis* adult abundance in a positive tendency (Figure 4 and Table 4), while relative humidity was negatively correlated with adult abundance and positively related to the nymphal peak (Table 3 and Table 4 and Figure 4). In contrast, larval activity did not correlate with any environmental variable (*p* > 0.05; Table 3).

Adults of *A. americanum* were occasionally found from March to the end of July (Figure 2b). Nymphs were consistently present from April 25 to the end of summer in September, although their numbers dramatically decreased in mid-July (Figure 2b). Lastly, larvae were present throughout the summer, from the end of July to the week of 22 September. The activity of the different tick stages of *A. americanum* did not correlate with environmental conditions (Table 3).

**Figure 5:**
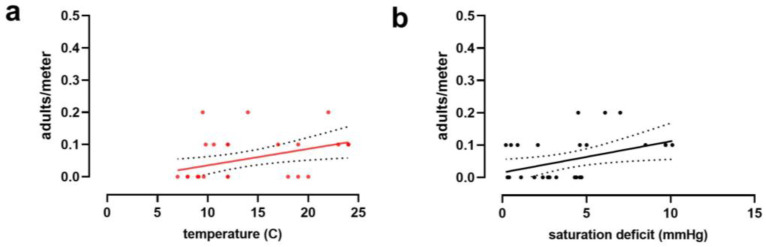
Representation of the linear regression equations between the environmental variables registered, temperature (**a**) and saturation deficit (**b**), and the relative abundance of *Ixodes scapularis* activity peaks of adults.

The majority of *I. scapularis* adults were collected in the fall on warm days (>10 °C), and occasionally in early spring during a few weeks between March and April (Figure 2c and Figure 3). Indeed, tick activity positively correlated with temperature and SD but not with relative humidity (Figure 5 and Table 3). On the other hand, nymphs remained active in the spring and early summer, from mid-April to the first week of July. However, they were sporadically observed in small numbers until November (Figure 2c), while larvae were active during the summer season from the first week of July to mid-September. The abundance of immature stages did not correlate with temperature, relative humidity, or SD (Table 3). Lastly, between mid-August and October, *I. scapularis* was rarely found in the ecotone (Figure 2c).

Adults of *D. variabilis* were sporadically found in the early spring and summer (Figure 2d), while immature stages were never collected. Due to the small number of individuals collected, correlation analyses with environmental variables were not performed.

## 4. Discussion

We found that ecotone habitats around and inside public parks and recreational areas in Middlesex (NJ) have a diverse population of ticks and their life stages. Significantly, we found that the invasive species *H. longicornis*, a tick identified in the US (NJ) in 2017 [29] is the dominant species in this habitat. However, other anthropophilic ticks, including *I. scapularis* and *A. americanum* are also present in high numbers. These tick species vector animal and human diseases and are actively present from the early spring until the fall, making the ecotone of public parks and recreational areas a high tick encounter risk area for humans and pets.

The seasonal dynamics of *H. longicornis* we found are similar to previous reports in a suburban area in NJ [23,30] and other eastern USA states [31,32,33,34]. However, the density of ticks in the ecotone in our study (1.7 ticks per m^2^) is higher than in other habitats [33,34] and like those found in previous studies conducted in ecotone habitats [23,30]. Collectively, these results indicate that *H. longicornis* is well adapted to edge habitats, which typically have drier conditions than forests [35]. This is not surprising since *H. longicornis* is often found in open meadows and forests in Asia and Oceania, suggesting that this tick species thrives in a wide range of temperature and humidity conditions [36,37,38]. In addition, biological factors, such as host availability, are critical for tick development and can drive important differences in tick densities [31,39]. For example, meso-mammals are the primary host for the immature stages in Asia, while in the USA, opossums and raccoons are the main potential hosts [40]. In contrast, small rodents do not seem to be important hosts for the immature stages [41], while deer and livestock can support all life stages [29,40]. Therefore, differences in *H. longicornis* densities may be driven by differences in the host communities between different habitats.

Despite the clear succession of *H. longicornis* stages throughout the year, there was considerable overlapping between life stages. This phenomenon has been described in Asia [38,42,43], Oceania [36], and, recently, in the USA [23]. The development stage sequence (nymph → adult → larva) is observed throughout the year with nymphs (spring) followed by adults (summer) and larvae (late summer and early fall), which suggests that the nymphal stage may be the main overwintering stage in NJ, as it was proposed recently for Virginia populations [44].

While *H. longicornis* is not an anthropophilic tick species, there are reports of human bites in the USA [45,46]. Furthermore, the presence of the tick in high numbers in highly urbanized areas and ecotone habitats suggests that the number of human and pet bites will likely increase in the following years. The public health impact of *H. longicornis* in the USA is still unclear. Vector competence experiments have shown that *H. longicornis* is not a competent vector of *Borrelia burgdorferi*—the agent of Lyme disease [47]; *Anaplasma phagocytophilum*—the agent of human granulocytic anaplasmosis [48]; or *Francisella tularensis*—the agent of tularemia [49]. In contrast, *H. longicornis* is a competent vector of *Rickettsia rickettsii*—the causative agent of Rocky Mountain spotted fever [50] and Heartland virus [51]. In addition, the Bourbon virus has been detected in field-collected specimens of *H. longicornis* [52].

More proximally, *H. longicornis* represents a novel threat from a veterinary standpoint as it frequently bites dogs [53,54] and can transmit *Babesia gibsoni*—one of the causative agents of canine babesiosis and an emerging pathogen in North America [55,56]. Coyotes can serve as hosts of *B. gibsoni* [57], and *H. longicornis* could mediate the enzootic transmission of the parasite between coyotes and stray dogs. Furthermore, the *H. longicornis* vectors *Theileria orientalis* Ikeda, an important parasite of cattle in New Zealand, Australia, and Japan [58]. This parasite has been detected in questing *H. longicornis* ticks in Virginia [59] and cattle [60]. Moreover, vector competence experiments have shown that USA *H. longicornis* ticks transmit *Theileria orientalis* Ikeda to calves [61].

Other tick species, including *I. scapularis* and *A. americanum*, were frequently found in the ecotone. The presence of anthropophilic ticks in edge habitats highlights the risk of tick encounters and transmission of tick-borne diseases in recreational areas and public parks. The spatial distribution patterns of *I. scapularis* have been studied in deciduous and coniferous woodlands. Some studies have found a greater abundance of *I. scapularis* in deciduous forests [62], while others showed a greater abundance in coniferous forests [8]. Nevertheless, it seems that the spatial distribution of *I. scapularis* is dynamic rather than dependent on the forest habitat and mediated by animal host movement [63]. Our results showed that *I. scapularis* is present in significant numbers in the ecotone. This result aligns with previous studies that showed that *I. scapularis* is commonly found in transitional brushy areas and edge habitats (ecotone) in the Northeast and upper Midwest [63,64,65].

Interestingly, the proportion of nymphs of *I. scapularis* in the ecotone differed from those observed for *H. longicornis* and *A. americanum*. While the proportion of nymphs of *H. longicornis* and *A. americanum* was roughly 10%, the proportion of nymphs of *I. scapularis* was 5%. Nymphs dwell in leaf litter and ground-level vegetation and have been reported to be more abundant in closed-canopy forests than in open habitats [66]. The leaf litter retains moisture, protecting nymphs from desiccation. In addition, leaf litter protects nymphs from harsh winter conditions, increasing their overwintering survivorship [67]. However, the leaf litter mass and coverage significantly decrease in the ecotone compared to the forest [68], which could explain the low proportion of nymphs compared to other life stages.

Instead, the proportion of adults in the ecotone is high compared to other tick species and higher than that of nymphs, suggesting that *I. scapularis* adults are well adapted to the ecotone. Moreover, the fact that adults are more abundant than nymphs suggests a tick influx most likely mediated by animals. Overall, the presence of *I. scapularis* in the ecotone highlights the importance of implementing tick-control programs in public parks and residential areas because it is the most important vector of human diseases in the northeastern USA [4].

The second most prevalent tick species in the ecotone was *A. americanum*, an aggressive biter responsible for most human bites in the USA [69,70] and NJ [71]. In a passive surveillance study conducted in NJ, most nymphs and adults of *A. americanum* were submitted during the spring and early summer [71], when these stages are most active in edge habitats. In the ecotone, larvae were only present in late summer, coinciding with the peak of larvae reported on hosts [72]. In NJ, all stages of this tick species are well adapted to different microclimate conditions [9], and therefore, it is not surprising to find them in ecotone habitats. However, it was less abundant than *H. longicornis* in our study. It is plausible that the invasive *H. longicornis* have displaced *A. americanum* in terms of relative abundance in ecotone areas of NJ due to their ability to reproduce asexually. In any case, the lone star tick is an important vector of human diseases and transmits the agents of human ehrlichiosis, *Ehrlichia chaffeensis* and *E. ewingii*, and tularemia, *Francisella tularensis* [73,74], and it is associated with the human alpha-gal “red meat” allergy [75].

Adults of *D. variabilis* are tolerant to desiccation and quest on open habitats, including meadows, ecotones, roadsides, and beachgrass [76]; however, only a few adults were collected in this study. In contrast, immatures were not found, which is expected since they are nidiculous [76,77]. Although *D. variabilis* vectors *R. rickettsii*, the agent of Rocky Mountain spotted fever, it does not seem to play a significant role in the transmission of this rickettsiosis in NJ [78]. Nevertheless, the presence of *H. longicornis*, which vectors *R. rickettsii* under laboratory conditions, and their ability to thrive in habitats similar to those exploited by *D. variabilis* could facilitate the transmission of *R. rickettsii* in enzootic cycles and increase the risk of human transmission.

Interestingly, the abundance of *H. longicornis* adults correlated with drier conditions, including higher temperatures, lower humidity, and higher saturation deficit. This result is remarkable because adults are active during the summer and, therefore, more active during hot summer days. In contrast, nymph abundance correlated with higher humidity and did not seem to be affected by temperature and SD. These results support that adults are the most drying-tolerant stage in the field. The only other stage for which we found an association with abiotic factors was *I. scapularis* adults. There was a correlation between abundance and temperature and SD. This is not surprising because adults, present in the fall and warmer days in this season, activate the questing behavior of the remaining adults in the field. It is well established that temperature and humidity can affect daily tick activity [79]. We did not look into changes in questing behavior throughout the day in this study that can be more dramatic than those recorded in this study.

**Figure 6:**
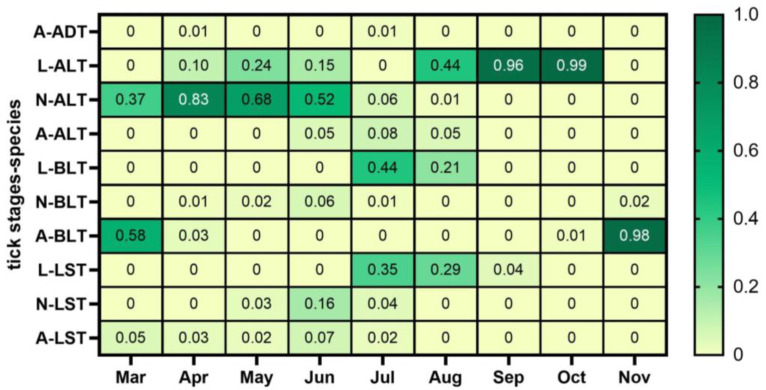
Map of the proportion of relative abundance observed of each stage and tick species in the ecotone. Life stages: larvae (L), nymphs (N), and adults (A). Tick species: *Dermacentor variabilis* (American dog tick, ADT), *Haemaphysalis longicornis* (Asian longhorned tick, ALT), *Ixodes scapularis* (blacklegged tick, BLT), and *Amblyomma americanum* (lone star tick, LST).

In summary, the ecotone of parks and recreational areas in central New Jersey is a suitable habitat for important tick vectors of human and animal diseases. Thus, tick surveillance in public parks and recreational areas is critical to determine the seasonal dynamics of tick species in edge habitats and inform stakeholders about their potential risk. Tick surveillance informs the public of the continuous risk that ticks represent for people and pets throughout the year and the importance of using personal protection measures. In addition, it can help park officials develop control and prevention plans to minimize the presence of ticks, including posting signals to alert the public of the presence of ticks, mowing the edge of trails, or implementing control in tick-infested areas that are heavily used by pets and people, among other measures. We have included a calendar with the proportion of each tick species and stage that could be useful for stakeholders in Middlesex (Figure 6).

## Figures and Tables

**Table 1:** Number of ticks per stage and species collected in ecotone areas from five sites in New Brunswick, NJ. Total proportion of each life stage is shown between parentheses for each tick species.

Life Stages	*Amblyomma* *americanum*	*Dermacentor* *variabilis*	*Haemaphysalis* *longicornis*	*Ixodes* *scapularis*	Total Ticks
Larvae	887 (86%)	0	8078 (89%)	647 (81%)	9612 (88%)
Nymphs	95 (9%)	0	819 (9%)	41 (5%)	955 (9%)
Adults	50 (5%)	10 (100%)	151 (2%)	106 (13%)	317 (3%)
Total	1032	10	9048	794	10,884

**Table 2:** Tick abundance in five different ecotone sites in New Brunswick, NJ. Kruskal–Wallis (KW) tests and ANOVA (F) were used to analyze significant differences between sites in total ticks and per tick species: *p* < 0.05 (*). Different letters represent significant differences using Dunn’s multiple comparisons tests (*p* < 0.05).

Site	*Amblyomma* *americanum*	*Haemaphysalis* *longicornis*	*Ixodes* *scapularis*	Total Ticks
1	0.022	0.039 ^a^	0.176 ^ab^	0.236
2	0.147	0.361 ^ab^	0.070 ^ab^	0.579
3	0.196	0.845 ^ab^	0.113 ^a^	1.154
4	0.101	0.459 ^ab^	0.004 ^b^	0.564
5	0.010	2.198 ^b^	0.026 ^ab^	2.238
Statistic *p*-value	3.90 (KW) 0.4196	10.45 (KW) 0.0335 *	10.43 (KW) 0.0338 *	1.79 (F) 0.1537

Data of total ticks were transformed (Log (Y + 0.1)) to normalize the distribution.

**Table 3:** Spearman rank (rs coefficient) correlations between the relative abundance of tick activity peaks and environmental variables. Life stages: larvae (L), nymphs (N), and adults (A). Asterisks represent a significant correlation (*p* < 0.05 (*), *p* < 0.01 (**)).

Tick Species	Life Stage	Peak of Tick Activity	N	Temperature			Relative Humidity			Saturation Deficit		
				rs	95% CI	*p*-Value	rs	95% CI	*p*-Value	rs	95% CI	*p*-Value
*Haemaphysalis* *longicornis*	L	18 August to 21 October	48	−0.092	−0.374 to 0.205	0.5332	−0.040	−0.329 to 0.255	0.785	−0.008	−0.299 to 0.285	0.956
	N	6 April to 16 June	40	0.004	−0.316 to 0.324	0.978	0.355	0.0395 to 0.606	0.024 *	−0.126	−0.429 to 0.202	0.439
	A	15 July to 1 September	40	0.351	0.035 to 0.603	0.026 *	−0.339	−0.595 to −0.021	0.032 *	0.369	0.056 to 0.617	0.019 *
*Amblyomma* *americanum*	L	29 July to 1 September	30	0.303	−0.075 to 0.605	0.104	−0.140	−0.485 to 0.242	0.459	0.207	−0.176 to 0.536	0.272
	N	3 May to 6 July	41	0.220	−0.103 to 0.502	0.166	0.251	−0.070 to 0.525	0.113	0.155	−0.169 to 0.449	0.332
	A	16 May to 2 July	30	−0.121	−0.470 to 0.261	0.525	−0.084	−0.440 to 0.295	0.659	−0.105	−0.457 to 0.275	0.580
*Ixodes* *scapularis*	L	6 July to 1 September	45	0.029	−0.275 to 0.328	0.849	−0.018	−0.318 to 0.285	0.908	0.004	−0.297 to 0.306	0.976
	N	3 May to 2 July	36	0.139	−0.208 to 0.455	0.420	0.112	−0.234 to 0.433	0.515	0.068	−0.276 to 0.396	0.693
	A	21 Oct to 24 November	30	0.549	0.225 to 0.764	0.002 **	−0.029	−0.395 to 0.345	0.880	0.377	0.008 to 0.655	0.040 *

**Table 4:** Simple linear regressions between the relative abundance of tick activity peaks (Y) and environmental variables (X). Asterisks represent a significant correlation (*p* < 0.05 (*)).

Environmental Variables		*Haemaphysalis longicornis* (N = 40)		*Ixodes scapularis* (N = 30)
		Nymphs	Adults	Adults
Temperature	Slope		0.007	0.005
	Std. Error		0.003	0.002
	R^2^		0.11	0.19
	F (DFn, DFd)		4.632 (1, 38)	6.686 (1, 28)
	*p*-value		0.038 *	0.015 *
Relative humidity	Slope	0.012	−0.002	
	Std. Error	0.005	0.001	
	R^2^	0.14	0.13	
	F (DFn, DFd)	6.132 (1, 38)	5.759 (1, 38)	
	*p*-value	0.018 *	0.021 *	
Saturation deficit	Slope		0.005	0.010
	Std. Error		0.002	0.004
	R^2^		0.14	0.17
	F (DFn, DFd)		6.283 (1, 38)	5.565 (1, 28)
	*p*-value		0.017 *	0.025 *

## Data Availability

Data are available from the authors upon request.
